# Bloodstream Infection Etiology among Children and Adults

**DOI:** 10.1155/2021/6657134

**Published:** 2021-03-01

**Authors:** Svetlana I. Kolesnichenko, Alyona V. Lavrinenko, Lyudmila L. Akhmaltdinova

**Affiliations:** Shared Resource Laboratory, Medical University of Karaganda, Karaganda 100008, Kazakhstan

## Abstract

Every year in the world, sepsis occurs in 31.5 million people, and the number of deaths reaches 5.3 million per year. There are not enough studies that describe etiological structure of sepsis pathogens in different groups of population of the Republic of Kazakhstan. In this study, we have investigated difference of local sepsis etiology and antibiotic susceptibility among children and adults. A total 200 blood samples were examined using the standard and express method of identification of bloodstream pathogens. The determination of antimicrobial sensitivity was carried out by the disc-diffusion method according to CLSI guidelines. Overall, 23/90 (25.5%) positive blood cultures were isolated from adult patients and 43/110 (39%) from pediatric patients. It was found that children are statistically more often affected with bacterial bloodstream infection than adults (*p* < 0.05). The Gram-positive bacteria are the leading cause of sepsis in both groups: *S. epidermidis* (35.5%) in pediatric patients and *S. aureus* (21.7%) in adults. However, statistical significance was detected in pediatric patients (*p* < 0.05). The number of resistant strains of *S. epidermidis* (MRSE) in the group of children was 66.7%, while in adults, all *S. epidermidis* was resistant to azithromycin and cefoxitin (MRSE). *S. aureus* strains from adult patients and children had a similar picture of antibiotic patterns. The proportion of MRSA in pediatric patients was 16, 6%, and in adult patients, 20%. Enterobacterales (39%) were the second cause of sepsis in adult patients. 62.5% of Enterobacterales strains isolated from adults were phenotypically identified as ESBL, while in pediatric patients, 25% of ESBL producers were isolated. We have noted the resistance to antibiotics that are prescribed according to protocols of treatment of the Republic of Kazakhstan in the strains isolated from the patient's blood.

## 1. Introduction

Sepsis is a life-threatening organ dysfunction which occurs as a result of a body's response to infection causes [1]. Every year in the world, sepsis occurs in 31.5 million people, and the number of deaths reaches 5.3 million per year [2]. To date, sepsis remains the main cause of mortality in children under 1 year of age. According to the WHO, about 3 million cases of sepsis among newborns and 1.2 million among children are detected annually in the world. The mortality rate from sepsis varies from 11% to 19% per 100,000 live births [3]. In the United States, for 72,000 hospitalized children diagnosed with sepsis, the mortality rate is 25% [4]. According to the SPROUT study, in 2013–2014, complications associated with sepsis in children ranged from 6% to 20% in Europe, South and North America, Australia/New Zealand, and Africa [5].

The primary foci of bloodstream infections are more often the lower respiratory tract in general [6], the respiratory system (40%), and the circulatory system (19%) in the pediatric departments [5]. According to Daneman et al.'s study [7], the most common sources of bacteremia in adults were the lung (27%), abdomen (25%), and urinary tract (23%).

Previous studies have found that the leading role in the bloodstream infections in adults belongs to microorganisms of the Enterobacterales family (*Escherichia coli* and *Klebsiella* spp.) and *Enterococcus* spp. [7]. If we talk about bloodstream infections in children, the data vary, according to a Yale-New Haven Hospital study for 2004–2013, *E. coli* was the cause of early sepsis in newborns in 45% of cases, followed by group B *Streptococcus* (36%) [8], and a similar situation is described by researchers from the United States [9, 10]. Other studies show that the leading positions are occupied by Gram-positive bacteria, coagulase-negative staphylococci (CoNS), *S. aureus* and *E. faecalis* (13%) [8]. In studies conducted in Southeast Asia, China, an equal distribution of Gram-positive and Gram-negative microorganisms in the etiology of blood stream infections in adults was noted, and Gram-positive pathogens dominated in children [11].

There are not much data on sepsis in the countries of the former Soviet Union: the epidemiology of sepsis in Russia can be judged from the Rudnov et al. multicenter study [6], where the prevalence of bloodstream infections in ICU was 34.1%. In the Republic of Kazakhstan, there are only a few local data, which do not reflect the entire epidemiological situation [12, 13].

Over the past 10 years, the advent of new technologies in microbiology has improved the diagnosis of septic conditions. The use of the direct detection method, using matrix laser desorption ionization time-of-flight mass spectrometry-MALDI-TOF, has become available for many laboratories. The simplicity of the preparation samples, the high quality of identification, time saving, and the low cost of analysis have made this method popular [14].

## 2. Objective

The objective of this study is to investigate difference of local sepsis etiology and antibiotic susceptibility among children and adults.

## 3. Materials and Methods

The study was conducted on the basis of the shared resource laboratory of the Medical University of Karaganda (Kazakhstan). Material for the study was blood samples from 200 patients diagnosed with sepsis. Patients were divided into 2 groups by age: children from 0 to 18 years old and adults from 18 to 99 years old. The average age of infants was 3.15 ± 2.49 months, and in children, 5.7 ± 5.3 years; in the group of adult patients, the average age was 51.6 ± 17.8 years (Table 1). Peripheral blood sampling was carried out directly in intensive care units from patients with aseptic technique in BD BACTEC™ Peds Plus vials before the beginning of antibiotic therapy. Blood was taken in a volume of 1–2 ml in children and adults. Within an hour from the time of blood sampling, the vials were delivered to the laboratory for further incubation using the BD BACTEC ™ FX blood culture system.

When a signal about a positive sample appears, 5 ml of broth is taken into serum gel-separator tubes. Gel-separator tubes were centrifuged on 3000 rpm for 10 minutes to separate media and bacteria from erythrocytes. Then, supernatant is discharged, and a thin white layer above gel is carefully mixed in 1-1, 5 ml ultrapure water, and moved into a 1, 5 ml Eppendorf tube. This sample is being centrifuged for 2 minutes on 13000 rpm. Supernatant is discharged, and the pellet is applied on target [15] and identified using the MALDI-TOF Microflex mass spectrometer (Bruker Daltonics, Germany). Identification was considered successful at the level of species with a high degree of confidence when the score exceeded 2.0; if the score was between 2.0 and 1.7, identification was considered successful at the genus level with adequate confidence [16].

Simultaneously, positive blood culture samples were cultured on blood agar with horse blood for 18–20 hours to exclude mixed cultures and to get isolated colonies for the antibiotic susceptibility test.

The determination of antimicrobial sensitivity was carried out for the prevailing microorganisms for each group of patients. Susceptibility of bacterial isolates to antibacterial drugs was carried out by the disk diffusion method to following antibiotics: penicillin G (10 u), ampicillin (10 *μ*g), cefepime (30 *μ*g), imipenem (10 *μ*g), meropenem (10 *μ*g), amikacin (30 *μ*g), gentamycin (10 *μ*g), netilmicin (30 *μ*g), tobramycin (10 *μ*g), nalidixic acid (30 *μ*g), ciprofloxacin (5 *μ*g), levofloxacin (5 *μ*g), linezolid (30 *μ*g), clindamycin (2 *μ*g), azithromycin (15 *μ*g), vancomycin (30 *μ*g), teicoplanin (30 *μ*g), fusidic acid (10 *μ*g), and tetracycline (30 *μ*g). Analysis of the results was carried out in accordance with the recommendations of CLSI 2018 [17]. Determination of bacterial isolate susceptibility to colistin was carried out by the broth microdilution method in accordance with the EUCAST guidelines [18, 19].

Phenotypic detection of methicillin resistant *S. aureus* (MRSA) and *S. epidermidis* (MRSE) was conducted by cefoxitin disk (30 *μ*g). MRSA positive strains had zone of inhibition ≤23 mm [17].

ESBL producers were determined by using cefuroxime (30 *μ*g), amoxicillin/clavulanic acid (20/10 *μ*g), and cefotaxime (30 *μ*g) disks [17].

Internal quality control was carried out on the control strains: *Staphylococcus aureus* ATCC 25923, *Pseudomonas aeruginosa* ATCC 27853, *Escherichia coli* ATCC 25922 (ESBL− and AmpC−), *Klebsiella pneumoniae* WHO-3 (ESBL +), and *Enterobacter cloacae* WHO-238 (AmpC + and ESBL−).

The analysis of susceptibility to antibacterial drugs was carried out by calculating the 95% confidence interval using the WHONET 5.6 program. Statistical analysis was carried out in the STATISTICA 7.0 program using the chi square.

## 4. Results and Discussion

Overall, a total 200 blood samples were processed. Of these, 23/90 (25.5%) positive blood cultures were isolated from adult patients and 43/110 (39%) from pediatric patients. It was found that bacteremia is statistically more often detected in children with sepsis than in adults (*p* < 0.05). From all positive samples, 4/66 (6%) were represented as mixed cultures (the associations of two microorganisms). It is useful to note that three of them were from patients up to 1 month of life. However, there is no statistical significance between frequency of mixed isolates and age of participants (*p* < 0.05).

The primary foci of infection in the examined patients were distributed as follows: in adult patients, the most common source of infection was the blood-vascular system (78.2%) and in pediatric patients, the respiratory system (48.6%) (Table 2).

**Figure 1:**
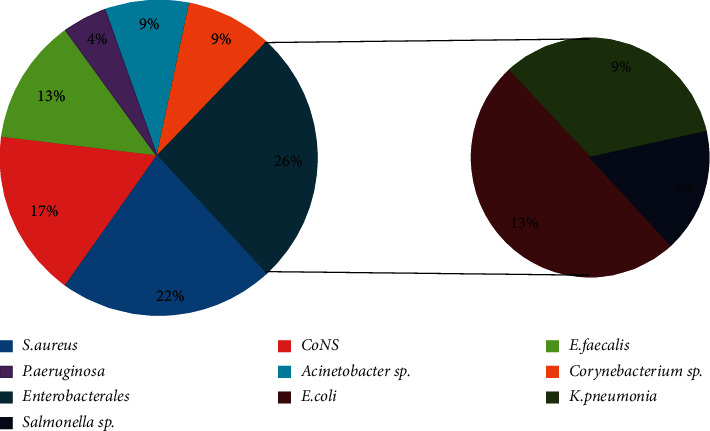
The causative agents of sepsis in adult patients.

**Figure 2:**
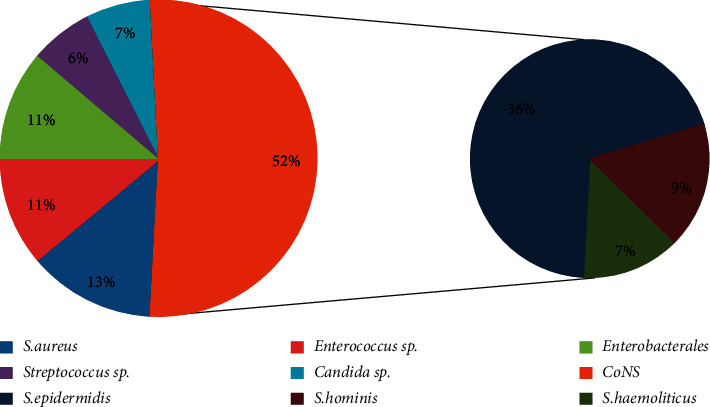
The causative agents of sepsis in children.

When examining the etiological structure of bloodstream infections, in the group of children, the most prevalence microorganism was *S. epidermidis* (35.5%) and in the group of adult patients, *S. aureus* (21.7%) (see Figure 1 and Figure 2). The Gram-positive bacteria play the leading role in patients of both groups. However, the ratio of Gram-positive and Gram-negative microorganisms in the children group was differed significantly: 37 : 5 (86% : 11.6%), so the Gram-positive bacteria in children were detected statistically more often (*p* < 0.05). In adults, the ratio of Gram-positive and Gram-negative bacteria is not much different, 14 : 9 (60.8% : 39.1%), and the second place after *S. aureus* in adults belongs to microorganisms of the Enterobacterales family (*E. coli* (13%), *K. pneumonia* (8, 7%), and *Salmonella* sp.(4, 3%)) (see Figure 1).

The study identified 3 cases of candidemia, and all of them were found in children. The causative agents were *C. albicans* and *C. parapsilosis*. According to past studies, the role of Candida fungi in the development of BSI has been marked among newborns and children of the first year of live much more often than among adults [20, 21].

**Figure 3:**
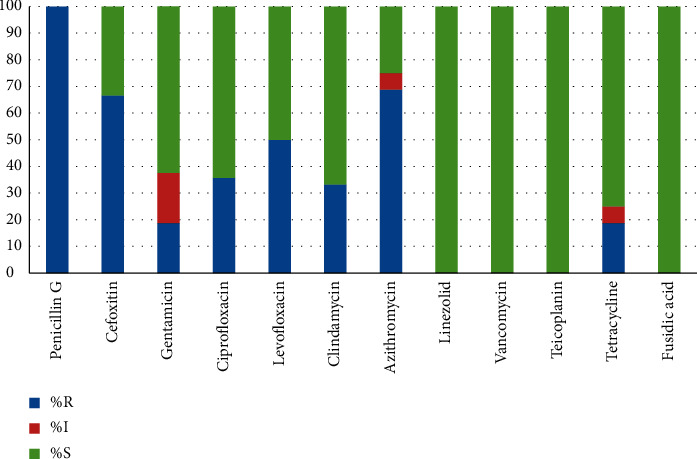
Sensitivity to antibiotics *S. epidermidis* from children.

During testing of the prevailing pathogens to antibiotics, we did not find trends of antimicrobial resistance among groups. The *S. epidermidis* strains isolated from the blood of pediatric patients were completely susceptible to penicillin, 100% (95% CI 74.7–100), 68.8% (95% CI 41.5–87.9) cases were resistant to azithromycin, 33.3% (95 % CI 13.0–61.3) cases were resistant to clindamycin, 35.7% (95% CI 14.0–64.4) were resistant to ciprofloxacin, and 50% (95% CI 20.1–79.9) were resistant to levofloxacin; to the group of aminoglycosides (gentamicin), 18 were resistant, 8% (95% CI 5.0–46.4) (see Figure 3). The proportion of MRSE was 66.7% (95% C.I. 38.7–87.0).

*S. epidermidis*, isolated from an adult group of patients, was characterized by a higher level of resistance to antibacterial drugs, and all strains were resistant to azithromycin and cefoxitin (MRSE). The remaining antibiogram data were similar to those in the group of children. All *S. epidermidis* strains isolated from adults and children were highly sensitive to vancomycin, linezolid, teicoplanin, and fusidic acid. The data obtained are consistent with the literature: in Amita Jain et al.'s study of catheter-associated bloodstream infections in the pediatric population, 63.1% oxacillin resistant CoNS and high glycopeptide sensitivity were obtained [22]; 66.6% of MRSE were found in a study of Santarpia et al. [23], but they also detected 55% of vancomycin-resistant strains. In our study, no vancomycin-resistant strains were detected, so it can be used to treat BSI caused by Gram-positive pathogens.

An important role in the etiology of sepsis in adults in our study was played by *S. aureus* (21.7%). *S. aureus* strains from adult patients and children had a similar picture of antibiotic patterns and were characterized by moderate sensitivity to antimicrobial agents. 1/5 (20%) of the strains from adults were MRSA; from pediatric patients, the proportion of MRSA was 1/6 (16.6%).

**Figure 4:**
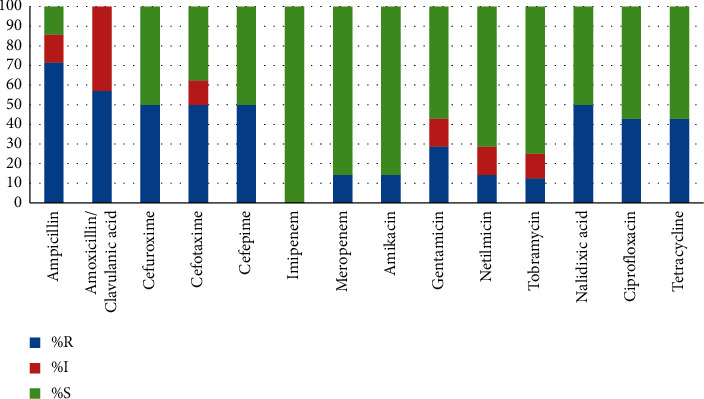
Sensitivity to antibiotic Enterobacterales strains from adults.

Enterobacterales (39%) were the second cause of sepsis in adult patients. 42.9% (95% C.I. 11.8–79.8) strains were resistant to fluoroquinolones (ciprofloxacin) and tetracycline while those from pediatric patients were highly susceptible, 100% (95% CI 0.0–60.4). Colistin (MIC> 2 100% (95% CI 0.0–37.1) and imipenem had high antibacterial activity in both groups. Meropenem was less active in adults, 14.3% of resistant strains. 28.6% (95% CI 5.1–69.8) of strains from adults were resistant to gentamicin, so as in children, 25% (95% CI 1.3–78.1). Other aminoglycosides in pediatric patients were completely susceptible (100%), while 12, 5% (95% CI 0.7–53.3), of isolates from adult patients were resistant to tobramycin and 14, 3% (95% CI 0.8–58.0), to netilmicin (see Figure 4). 62.5% of Enterobacterales strains isolated from adults were phenotypically identified as ESBL, while in pediatric patients, were isolated 25% of ESBL producers. In a study by Abdallah H. M., 48.93% of ESBL producers were isolated from patients with a bloodstream infection, which is consistent with our data [24]. Similar results were obtained in China, and 67.8% of ESBL producers were isolated from positive blood samples [25].

## 5. Conclusions

In the results of our study, we found that bacteremia is statistically more often detected in children with sepsis than in adults (*p* < 0.05). The most frequently isolated microorganisms in both groups were Gram-positive cocci: in children, *S. epidermidis* (35.5%); in adults, *S. aureus* (21.7%). The Gram-positive bacteria in children were detected statistically more often (*p* < 0.05) than Gram-negative with ratio 37 : 5 (86% : 11.6%). The number of resistant strains of *S. epidermidis* (MRSE) in the group of children (66.7%) significantly exceeds the number of those in adults, which may be due to the localization of the primary focus of infection and more frequent administration of antistaphylococcal drugs. In adult patients, a large percentage of ESBL (62.5%) relative to children (25%) draws attention, which is associated with the spectrum of prescribed antibacterial drugs according to the protocols of diagnosis and treatment of the Republic of Kazakhstan.

## Figures and Tables

**Table 1:** Characterization of patients by groups.

Group of patients	Age	Mean age ± SD	Total examined	Number of “+” samples
Children	0–12 month	3.15 ± 2.49	57	43 (39%)
	1–18 years	5.7 ± 5.3	53	

Adults	18–99	51.6 ± 17.8	90	23 (25.5%)

**Table 2:** Primary foci of infection in patients with sepsis.

Primary foci	Children (*n* = 43) (%)	Adults (*n* = 23) (%)
Respiratory tract	48, 6	13
Blood-vascular system	—	78, 2
Gastrointestinal tract	23, 2	4, 3
Skin-soft tissue/bone-joint	9, 3	—
Intranatal	11, 6	—
Central nervous system	6, 9	—
Genital tract	—	4, 3

## Data Availability

The data used to write this publication are the property of the Ministry of Education and Science of the Republic of Kazakhstan and are available from the corresponding author upon request.
